# 3D strain pattern in additively manufactured AlSi10Mg from digital volume correlation

**DOI:** 10.1016/j.heliyon.2023.e23186

**Published:** 2023-12-03

**Authors:** Xinyang Gao, Yubin Zhang, Lasse Haahr-Lillevang, Nikolaj Kjelgaard Vedel-Smith, Tito Andriollo

**Affiliations:** a Department of Civil and Mechanical Engineering, Technical University of Denmark, DK-2800, Kgs. Lyngby, Denmark; b Danish Technological Institute, DK-8000, Aarhus, Denmark; c Department of Mechanical and Production Engineering, Aarhus University, DK-8000, Aarhus, Denmark

**Keywords:** Laser-based powder bed fusion, X-ray tomography, Digital volume correlation, Strain, AlSi10Mg, Heterogeneities

## Abstract

Although much research has focused on AlSi10Mg processed via laser-based powder bed fusion, the material deformation mechanisms at the microscale are still unclear. To improve the current understanding, 3D measurements of the strain field at the microstructural scale are needed to complement surface-based SEM observations. This work demonstrates that X-ray tomography combined with digital volume correlation can be used to measure the strain in the bulk of AlSi10Mg using the Si-rich particles contained in the heat-treated microstructure as markers. The method allows for measuring strains larger than 0.5 % with a spatial resolution of 35 μm and it can thus be used to study the impact of factors like porosity distribution or crystallographic texture on the material deformation and damage mechanisms.

## 1 Introduction

Extensive research is being devoted to studying AlSi10Mg processed via laser-based powder bed fusion (PBF-LB). The primary reason is the good material processability combined with the possibility of achieving attractive combinations of tensile strength, ductility and fatigue resistance by heat-treating the as-printed microstructure [[1], [2], [3]]. Most heat-treatment strategies produce microstructures consisting of globular Si-rich particles embedded in Al-rich matrices [4,5]. Although the global mechanical properties of such microstructures have been investigated, the mechanisms associated with their deformation and damage at the microscale still need to be clarified [3,5]. In this respect, past research efforts have mainly relied on in-situ SEM as the investigation method [[6], [7], [8], [9]]. However, in a material containing particles the local stress state on the specimen surface can differ substantially from that in the bulk [10]. Consequently, 3D investigations are necessary to complement the SEM findings.

In-situ X-ray tomography can be a valuable tool for the 3D characterization of PBF-LB-processed AlSi10Mg. The technique was already employed to monitor the growth of sub-surface pores [7]. Yet, its potential can be greatly enhanced by coupling it with digital volume correlation (DVC) [11,12], which allows for reconstructing the 3D strain field over a material volume by using the microstructural features appearing in the tomogram as markers. Pores are clearly distinguishable in a tomogram of heat-treated AlSi10Mg, but their low volume fraction – typically limited to 1 % or less – prevents using them for DVC purposes. As an alternative, we investigate in this paper the possibility of imaging the Si-rich particles via lab X-ray tomography and using them, instead of the pores, as markers for DVC.

## 2 Materials and methods

**Fig. 1:**
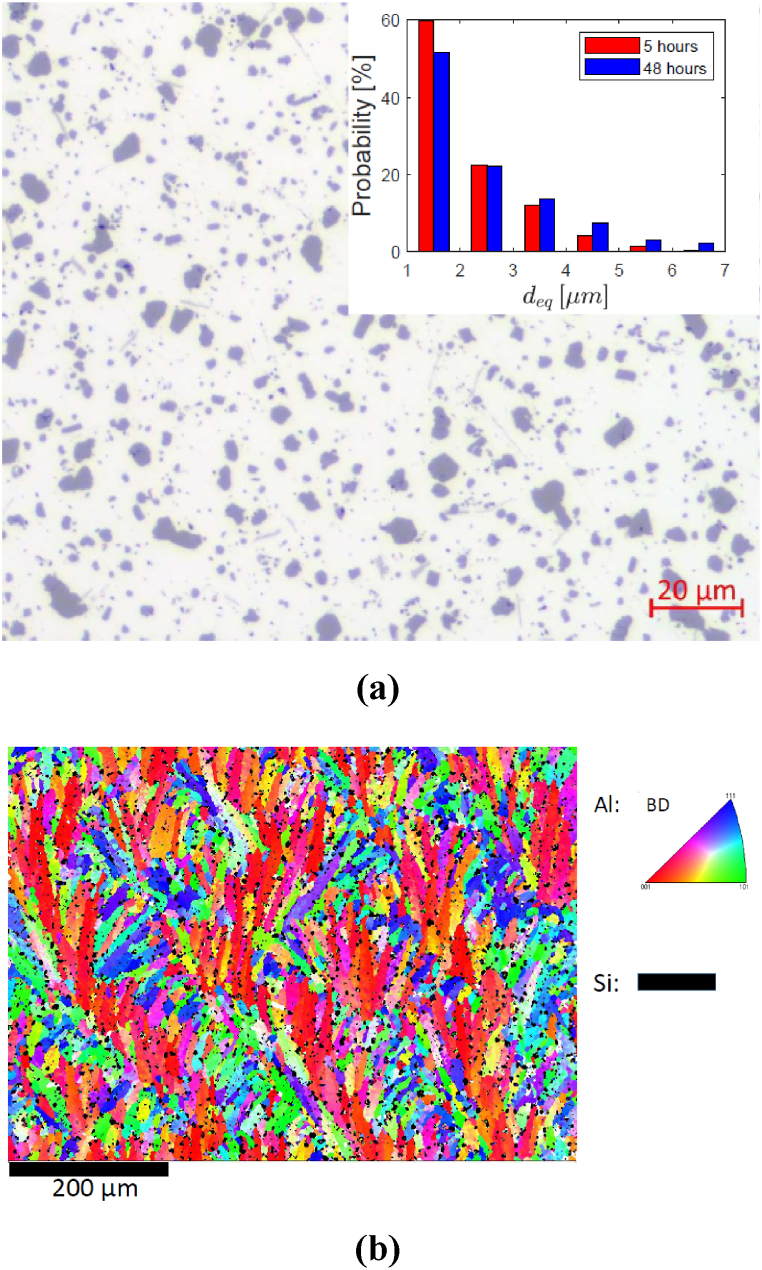
Microstructure of AlSi10Mg after annealing at 520 °C for 48 h. (a) Optical microscope image. The inset compares the distribution of the particle equivalent diameter with that of AlSi10Mg annealed at the same temperature for 5 h. (b) EBSD map. The build direction is vertical.

Blocks of AlSi10Mg with size 30 × 20 × 10 mm^3^ were manufactured with PBF-LB using layer thickness 60 μm, hatch distance 170 μm, power 650 W and laser speed 1850 mm/s. The build direction was set parallel to the long side of the blocks and a stripe scanning strategy was adopted, with each layer rotated by varying angles. The as-printed blocks were then annealed at 520 °C, for either 5 h – which is common practice [13] – or 48 h, to create larger particles that are easier to image with X-rays. Fig. 1 (a) shows the microstructure of a block annealed for 48 h, where the dark Si-rich particles are distinguishable from the lighter Al-rich matrix. The figure inset compares the distribution of the particle equivalent diameter with that of the block annealed for 5 h. The equivalent diameter is computed based on the area of each particle and values below 1 μm are not reported, as the interest is on the larger particles that might act as effective markers for DVC. It can be noticed that the 48-h treatment leads to a substantial increase in the number of particles with sizes above 3 μm compared to the 5-h treatment (+160 %). By contrast, Fig. 1 (b) shows that the long annealing time has little effect on the grain structure, which is similar to that of the material in the as-printed condition [7].

**Fig. 2:**
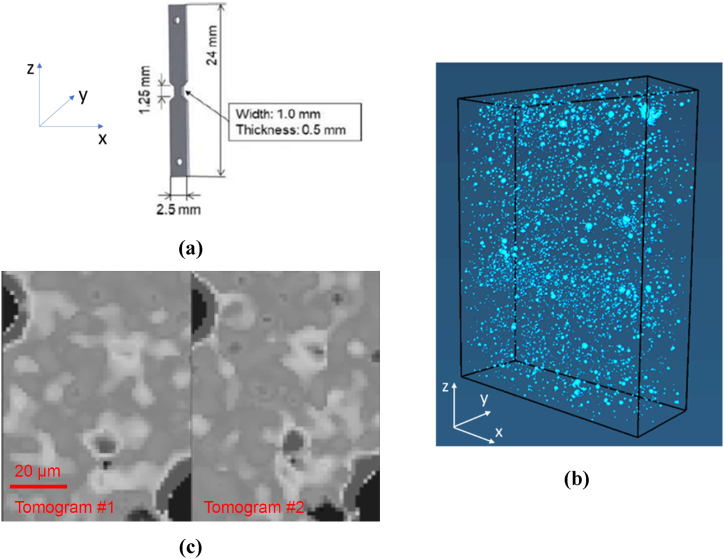
In-situ X-ray imaging. (a) Geometry of the tensile specimen with indication of the adopted coordinate system. (b) Reconstructed pores in the 600 × 200 × 750 voxel^3^ ROI defined in the center of the specimen. (c) Slices of separate tomograms corresponding to the same physical location. The gray scale is the same in both tomograms and represents the level of X-ray attenuation (white = max attenuation, black = min attenuation).

A specimen with the geometry indicated in Fig. 2 (a) was extracted from the 48-h- annealed block and mounted on a displacement-controlled tensile stage inside a Zeiss Xradia 520 Versa CT system. The specimen was initially scanned in the unloaded configuration, setting the acceleration voltage to 40 kV and electric power to 2 W. A total of 1801 projections were acquired during a full 360° rotation of the specimen, using an optical magnification of 4X and an exposure time of 55 s. The source-to-sample and sample-to-detector distances were 13.5 mm and 55 mm, respectively. A Feldkamp reconstruction algorithm [14] for cone beam reconstructions was applied, resulting in a reconstructed 3D tomogram with a voxel size of 1.42 × 1.42 × 1.42 μm^3^ under a 2 × 2 detector binning.

**Fig. 3:**
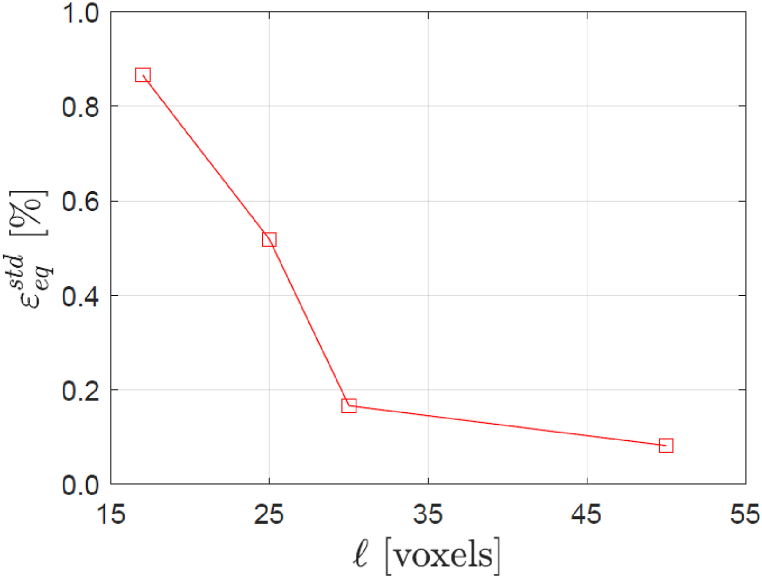
Effect of the subset size l on the strain measurement resolution, expressed as the standard deviation $\varepsilon_{eq}^{std}$ of the von Mises equivalent strain computed considering the two scans of the specimen in the unloaded configuration.

As previously mentioned, the identification of the porosity in a tomogram of AlSi10Mg is straightforward, due to its negligible level of X-ray attenuation. Accordingly, Fig. 2 (b) shows the reconstructed pores within a 600 × 200 × 750 voxel^3^ region of interest (ROI) located in the center of the specimen. It can be noticed that the pores appear unevenly distributed in space. That is, local fluctuations of their volume fraction compared to the mean value – which is about 1 % – seem to exist. Nevertheless, the pores are not the only microstructural features that are captured by the X-rays. To demonstrate this, a second tomogram was acquired with the same settings, still without applying any loading to the specimen. Fig. 2 (c) compares slices of the two tomograms from the same physical location in the specimen. Two pores are clearly visible in black in the top-left and bottom-right corners. In addition, several other features can be distinguished between these two pores, most of which are present in both tomograms and therefore cannot be ascribed to noise. The attenuation contrast is not sufficient to segment such features unambiguously, but their size and spacing suggest, in the light of the microstructure reported in Fig. 1 (a), that they are associated with the Si-rich particles. To investigate whether they can be employed as markers for DVC, the subset-based DVC algorithm of the software Avizo® was used to compute the strain field associated with the mapping of the material points in the ROI from one tomogram to the other. Since no deformation was applied to the specimen, the strain standard deviation can provide an estimate of the measurement resolution [15]. Fig. 3 shows how the standard deviation of the equivalent von Mises stress is affected by the choice of the subset size. It can be seen that by selecting a subset size of 25 voxels, the standard deviation is ≈ 0.5 %, suggesting that strains larger than this value can be measured using the abovementioned microstructural features as markers.

The tensile specimen was then deformed up to a nominal strain of 2 % and a third tomogram was acquired. Subset-based DVC was performed using this third tomogram as the deformed input image and the very first tomogram as the reference input image. To improve the convergence of the DVC algorithm the specimen ROI was subdivided into 10 subregions with dimension 300 × 200 × 150 voxel^3^ each. DVC was performed at the level of each subregion using 25 voxels as subset size, and the output was eventually combined to obtain strain data covering the entire ROI.

## 3 Results and discussion

**Fig. 4:**
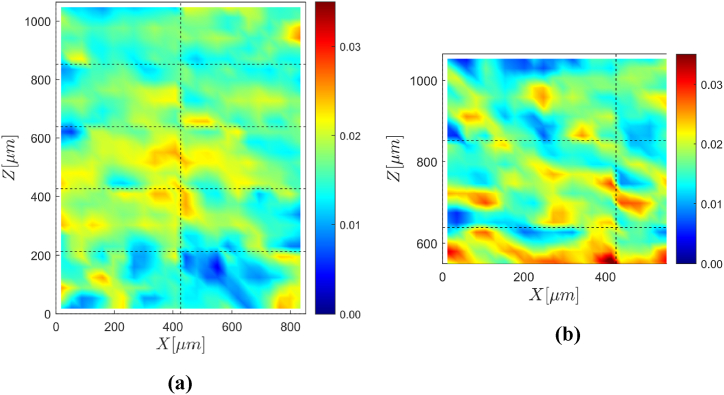
Strain pattern measured with DVC. (a) Contour of the equivalent strain over the middle X-Z section of the specimen ROI. (b) As in (a) but limited to the top-left portion of the X-Z section and computed using a smaller subset size of 17 voxels.

Fig. 4 (a) shows the equivalent strain computed with DVC over the middle X-Z section of the ROI. The Z-axis is aligned with the direction of tensile loading, which coincides with the build direction. Despite the long annealing treatment, the deformation pattern appears highly heterogeneous at the microstructural scale. Shear bands oriented at ≈ 60° with respect to the loading direction are clearly distinguishable in some areas. This is especially evident in Fig. 4 (b), which shows the strain pattern corresponding to the top-left area of Fig. 4 (a) computed with higher spatial resolution, i.e., using a DVC subset size of 17 voxels instead of 25 voxels. Three facts support the trustworthiness of this strain pattern. First, the shear bands in Fig. 4 (b) are distinguishable in Fig. 4 (a) as well, meaning that the main features of the strain pattern are independent of the choice of the DVC subset. Second, the shear bands are continuous across the boundaries of the subregions used as independent DVC calculation domains – marked by black dashed lines in Fig. 4. Consequently, the shear bands cannot be DVC artifacts. Finally, the mean value of the zz-component of the strain over the entire ROI is +1.8 %, which agrees with the nominal tensile deformation of 2 % imposed on the specimen.

**Figure (5):**
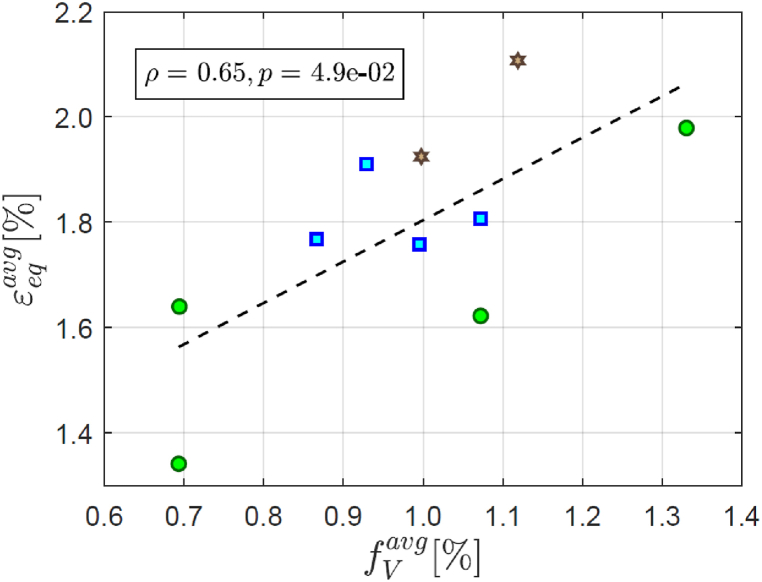
Mean equivalent strain in each subregion as a function of the corresponding mean porosity volume fraction. Datapoints corresponding to subregions located symmetrically about the specimen center are reported using the same marker. The dashed line corresponds to a linear fit.

It is known that AlSi10Mg in the as-printed condition exhibits a microstructural damage mechanism that does not involve formation of shear bands [6]. Conversely, the strain pattern for the heat-treated AlSi10Mg considered in this study presents shear bands that somewhat resemble those observed in porous metals [16]. This fact suggests that the shear bands might be caused by the pores shown in Fig. 2 (b). Fig. 5 reports the mean porosity volume fraction $f_{V}^{avg}$ and the mean equivalent strain $\varepsilon_{eq}^{avg}$ computed at the level of each of the 10 subregions the ROI was subdivided into, where the same marker is used for subregions that are located symmetrically about the specimen center. If the porosity didn't play a role at all, the regression line should have a slope close to zero. This is, however, not the case. Accordingly, the values of the Spearman's rank coefficient and its *p*-value – reported in the figure – indicate a significant positive correlation between $f_{V}^{avg}$ and $\varepsilon_{eq}^{avg}$. Although these facts suggest that the pores affect the strain locally, they are not sufficient to conclude that the shear bands are caused by pores. The main value of Fig. 5 lies, however, in showcasing the type of analyses that are enabled by the 3D strain measurement method presented in this article. In fact, the porosity influence on plastic deformation is expected to be dependent on the stress triaxiality [17], which varies between the bulk and the specimen surface and therefore cannot be studied solely using 2D techniques.

## 4 Conclusions

The present study demonstrates that lab X-ray tomography combined with DVC allows to measure the 3D strain field in the bulk of heat-treated AlSi10Mg by using the Si-rich particles as markers. This will enable future investigations into how microstructural features like porosity, crystallographic texture, etc. affect the material bulk deformation and damage mechanisms.

## Funding

This work received support from the 10.13039/100008393Danish Research Council for Independent Research, grant no. 2035-00142B.

## CRediT authorship contribution statement

**Xinyang Gao:** Formal analysis, Investigation. **Yubin Zhang:** Investigation, Supervision, Writing – review & editing. **Lasse Haahr-Lillevang:** Resources, Writing – review & editing. **Nikolaj Kjelgaard Vedel-Smith:** Resources, Writing – review & editing. **Tito Andriollo:** Conceptualization, Formal analysis, Supervision, Writing – original draft.

## Declaration of competing interest

The authors declare that they have no known competing financial interests or personal relationships that could have appeared to influence the work reported in this paper.
